# Framework for charged compact objects admitting conformal motion in higher dimension

**DOI:** 10.1371/journal.pone.0307489

**Published:** 2024-08-26

**Authors:** A. Zahra, S. A. Mardan, Muhammad Bilal Riaz, Rubab Manzoor

**Affiliations:** 1 Department of Mathematics, University of the Management and Technology, Lahore, Pakistan; 2 IT4Innovations, VSB-Technical University of Ostrava, Ostrava, Czech Republic; 3 Department of Computer Science and Mathematics, Lebanese American University, Byblos, Lebanon; University of Sharjah, UNITED ARAB EMIRATES

## Abstract

We propose a new framework for spherical charged compact objects admitting conformal motion in five-dimensional spacetime. The outer spacetime is considered as Reissner-Nordström to obtain matching conditions. The behavior of model characteristics like stress, pressure, and surface tension for the specific density profile is investigated by using Einstein’s Maxwell field equations in a five-dimensional framework. For the proposed solution, all physical parameters behave very well even for variations in electric charge parameters. The existence of charged compact stars is also predicted by this study.

## Introduction

In general relativity (GR), the Einstein-Maxwell field equations (EMFEs) are used to analyze charged relativistic objects. This analysis is very challenging due to the nonlinearity of the EMFEs and the general exact solution is nearly impossible to obtain. So different authors used various constraints in relativistic astrophysics to obtain restricted solutions. The interesting results employing conformal killing vectors (CKV) were presented in [1–3]. Mak and Harko [4] developed a precise solution that reflected a strange quark star with charge. When the space–time configuration is taken to be spherically symmetric, their solution describes the interior of the star. Esculpi and Alomá [5] investigated the conformal anisotropic relativistic charged fluid spheres using a linear equation of state. Shamir [6] discussed the massive charged compact Bardeen stars with conformal motion. Lemos and his coworkers [7–9] studied the regular black hole solutions are obtained within general relativity connected to Maxwell’s electromagnetism and charged matter, and they also examined the quasi-black hole solutions from extremal charged dust. Charged radiating sphere with anisotropic fluid have been investigated by many authors [10, 11]. Thirukkanesh and Maharaj [12] used the linear equation of state to examine the strange stars with anisotropy and electric charge.

The EMFEs are the cornerstone of the general theory of relativity as they link geometry and matter in space-time configuration. One approach to obtaining EMFEs solutions is to impose some symmetry requirements. These symmetry constraints play a vital role in the theory of GR since they are very useful in determining the solutions of EMFEs. Killing symmetry, characterized by the vanishing of the Lie derivative of a metric tensor, stands as a fundamental and significant form of symmetry.

On the other hand, inheritance symmetry is useful for investigating the natural relationship between geometry and matter using EMFEs. The symmetry under CKV is a well-known inheritance symmetry that can be expressed as

$$\begin{array}{c} L_{\xi }g_{mn}=\psi g_{mn},\;\;\;\;\;m,n=1,2,3,4,5, \end{array}$$ (1)

where *L* denotes the Lie derivative of the metric tensor *g*. The relationship between the vector field *ξ* and the inner gravitational field of a compact objects (CO) is shown by this equation. Conformal symmetry is characterized by the vector *ξ*, enabling the metric *g* to undergo conformal translations along *ξ* and map onto itself. The conformal factor, denoted as *ψ*, is a function that depends on the variable *r* and has the freedom to vary in any manner. Therefore, in a static metric, it’s noteworthy that both *ξ* and *ψ* aren’t mandated to remain static [13, 14]. If *ψ* = 0, Eq (1) yields the Killing vector, which is homothetic when *ψ* is constant. The existence of *ψ* = *ψ*(*x*, *t*) enables the construction of a conformal vector [15]. The study of CO makes extensive use of conformal motion. The anisotropic matter is studied for the existence of a one-parameter group of conformal motions. The analysis shows that the EMFEs clearly define the equation of state for both isotropic and anisotropic matters. Moreover, since the positive energy densities of both solutions exceed the stresses across the whole spherical region, they are both consistent with the Schwarzschild exterior metric [16]. Conformal motions are employed to address the EMFEs linked to spherically symmetric configurations of both isotropic and anisotropic matter. Solutions with a surface potential of 1/3 align with the Schwarzschild exterior metric. The third set of solutions characterizes oscillating black holes, whereas the initial two sets elucidate the dynamics of expanding and contracting spheres. Each solution exhibits a density profile surpassing the stress level [17, 18]. The discussion of a one-parameter group with conformal motions and spherical symmetry has resulted in analytical solutions for static EMFEs in both perfect and anisotropic fluids. These solutions show positive energy density all the time when they are coupled to the Reissner-Nordström metric, and they exceed the total stresses of the sphere [19]. There are many techniques to solving the EMFEs for an anisotropic matter distribution with spherical symmetry. These solutions coincide with Minkowski spacetime at the boundary, despite the pressures and energy densities remaining within the sphere [20]. Anisotropic fluids admitting conformal collineation, a generalization of conformal motion, are addressed in GR [21]. The findings of the research gave rise to spacetimes with a conformal motion group on *S*^2^ that has just one parameter and is axially and reflection symmetric. For the Einstein vacuum field equations, Minkowski represents the only physically meaningful solution [22].

Kinematics and dynamics in GR are defined by variables and constraints. A significant limitation pertains to symmetry, which can be classified into two groups: killing vectors and curvature collineations. The EMFEs for general spacelike collineations and matter are made simpler by using the CKV. Applications to spacelike CKV and matter characterized by perfect or anisotropic fluids are considered. We demonstrate that matter does not inherit symmetry [23], by extending the concept of symmetry inheritance to CKV. Within the thin-wall approximation, two groups of time-dependent EMFEs solutions characterize scalar soliton stars. Second-order phase transitions emerge due to the self-similarity of space-time within stars. Di Prisco [24] furnishes details about the minimum and maximum surface potentials involved in this phenomenon. In order to explore all conceivable evolution scenarios, the differential equation governing the time evolution of self-similar scalar soliton star models, as mentioned in [25], is contrasted with specific solutions. Specific situations are unified and generalized by the conformal Killing equation of static spherically symmetric spacetimes.

In a one-parameter spherically symmetric spacetime, they investigated the structure for conformal motions of charged strange quark matter coupled to a string cloud. To do this, EMFEs for spherically symmetric spacetime are solved taking into consideration the conformal motions used by strange quark matter to cling to the string cloud. They also explore the properties of the obtained solutions [26]. They carry out a general examination using a CKV into static fluids that are axially symmetric. This kind of symmetry is highlighted in terms of its physical significance. After that, they examine every potential consequence that could result from implementing this kind of symmetry. A specific treatment is given to the problem of symmetry inheritance [27]. Exact analytical solutions are derived for dynamic spherically symmetric fluids with CKV, considering various CKV possibilities in both adiabatic and dissipative regimes. The study examines various methods for identifying novel approaches and whether they are appropriate for outcomes in astrophysical settings [28]. Herrera et al. recently showed in their work [16] that when the resulting CKV field is orthogonal to the four velocities, the equation of state *p* = *ρ* is uniquely determined for particular conformal motions. They modify this obstacle and show that there is no particular conformal collineation for which the equation of state is intended to [29]. Ray et al. [30] explored the electromagnetic mass model admitting conformal motion and analyzed the several features of COs. Herrera et al. [28] studied non-static spherically symmetric fluids with a conformal Killing vector revealing several exact analytical solutions for different CKV choices in dissipative adiabatic regimes. They discussed the applications and alternative approaches to find solutions. Pradhan and Sahoo [31] studied the massive compact star admitting conformal motion under bardeen geometry. Their analysis reveals that the resulting compact star solutions are physically acceptable and authentic when considering the presence of charge with conformal motion in *f*(*Q*) gravity.

In recent years, the theory of GR has played a vital role in higher dimensions. In order to evaluate the viability of gravitational redshift, light bending, perihelion shift, and gravitational time delay, Rahaman et al. [32] examined four solar system experimentation scenarios. Further investigation into the motion of a test particle in greater dimensions was carried out by Liu and Overduin [33]. Rahaman et al. [34] study D-dimensional gravastar. A new class of four-dimensional interior solutions for anisotropic compact stars was studied by Rahaman et al. [1]. They [35] also used noncommutative geometry with a Gaussian energy density distribution to study fluids in both the higher and lower dimensions. They proved that there is only one stable four-dimensional configuration for a spherically symmetric star system. Zahra et al. [36] studied the conformal motion for higher dimensional COs. Bhar et al. [37] provided evidence for the existence of higher dimensional anisotropic compact stars in noncommutative space-time. They found that the physical behaviors of the model parameters, such as matter energy density, radial and transverse pressures, anisotropy, and other characteristics, are generally consistent throughout the stellar structure. They also mention that as one goes to higher dimensions, the central densities abruptly decrease, and that the measure of anisotropy gradually increases, reaching its maximum at five dimensions. A star’s central density is greatest in four dimensions and lowest in higher dimensions.

Many researchers studied higher dimensional gravity theory and motivated us to explore more about higher dimensional charged COs admitting conformal motion. Chattopadhyay [38] and his coworkers discussed the relativistic charged star solutions in higher dimensions and found that dependence of the central density on space-time dimensions enables a practical model for *D* ≥ 4. The significance of the strong energy and weak energy conditions is investigated for COs. New solutions with captivating stellar models are discovered, allowing for stars with varying core properties. Dey and Paul [39] examined relativistic solutions with Finch-Skea geometry of anisotropic charged COs with hydro-dynamical equilibrium in higher dimensions. By establishing physically feasible stellar models with these solutions, radial variations in density, pressure, and other physical parameters are investigated. The findings could be useful in predicting equation of state under extreme conditions and in comprehending the physical characteristics of known stars.

Numerous investigators come to the conclusion that the (Tolman–Oppenheimer–Volkoff) TOV equation is satisfied by a homogeneous, isotropic, or perfect fluid which supports the CO underlying matter distribution [40, 41]. This method is widely used in the description of CO, especially neutron stars and polytropic CO [42]. Recent theoretical developments show that pressure anisotropy can arise from several sources and that pressure within a CO does not have to be entirely isotropic [43]. In actuality, a great deal of research has been done on anisotropic fluids in the past with the goal of identifying causes of sustained anisotropy. [44] offers a comprehensive description of anisotropic fluids as well as a long list of possible physical events that could be involved in the emergence of pressure anisotropy. As shown in [45], we examined the five-dimensional dynamic spherically symmetric distribution with a particular energy density profile. Bhar et al. [46] investigated the effect of *f*(*Q*) gravity on an anisotropic compact star model and stability study of hybrid star models. Rashid et al. [47] explored Bardeen stars with conformal motion under *f*(*G*) gravity. They discovered that COs are physically viable and stable under conformal motion in the conditions of Bardeen geometry for different models of the *f*(*G*) theory of gravity. They suggested that using the Bardeen model with *f*(*G*) gravity can result in more massive star objects than general relativity. Asghar et al. [48, 49] examined the embedded class-I fluid spheres in *f*(*R*, *ϕ*) and *f*(*R*, *T*) gravity with the Karmarkar condition. The articles [50–54] investigated the modeling of COs in the framework of modified gravity theory. These articles are possible extensions to this existing work.

In this article, we will show that CO also exists in the five-dimensional non-static charged spherical symmetric spacetime. An outline for this article is as follows: We provided the EMFEs for the internal spacetime of the charged isotropic star in Section 2. Section 3 solves the EMFEs using the specific density profile given in Equation ([1]). We provided the five-dimensional exact solution for the physical parameter analysis in Sect. 4. The external spacetime and charge-induced anisotropy are presented in Section 5. In Sect. 6, we examined the properties of the model, the stability requirements, and the fifth-dimension graphical plot. We present our final remarks in Sect. 7 to conclude the paper.

## The higher dimension EMFEs

The five-dimensional line element is assumed to be dynamic spherically symmetric spacetime as follows [55]:

$$\begin{array}{c} ds^{2}=e^{\nu (r,t)}dt^{2}-e^{\lambda (r,t)}dr^{2}-r^{2}\left(d\theta^{2}+sin^{2}d\phi^{2}\right)-e^{\mu (r,t)}dw^{2}, \end{array}$$ (2)

The spacetime coordinates are *x*^0^ = *t*, *x*^1^ = *r*, *x*^2^ = *θ*, *x*^3^ = *ϕ*, *x*^4^ = *w*. The components of the energy-momentum tensor for the matter source and the electromagnetic field are provided as follows:

$$\begin{array}{c} T_{\nu }^{\mu (m)}=\left(\rho +p\right)u^{\mu }u_{\nu }-pg_{\nu }^{\mu }, \end{array}$$ (3)

$$\begin{array}{c} T_{\nu }^{\mu (em)}=\frac{1}{4\pi }\left[F_{\nu k}F^{\mu k}-\frac{1}{4}g_{\nu }^{\mu }F_{\sigma k}F^{\sigma k}\right]. \end{array}$$ (4)

Here *ρ*, *p*, and *u*^*μ*^, represent the density, pressure, and fluid five velocity (with $u^{\mu }=e^{-\nu /2}\delta_{0}^{\mu })$ respectively. The corresponding Maxwell electromagnetic field equations are

$$\begin{array}{c} {\left(\sqrt{-g}F^{\mu \nu }\right)}_{,\nu }=4\pi \sqrt{-g}J^{\mu }, \end{array}$$ (5)

$$\begin{array}{c} F_{\left[\mu \nu ;\sigma \right]}=0, \end{array}$$ (6)

where the expression *J*^*μ*^ = *σu*^*μ*^ denotes the five-vector for electric current, where *σ* represents electrical conductivity. In this context, the symbols “,” and “;” signify partial differentiation and covariant derivative concerning the specified coordinate, respectively.

The EMFEs in five dimensions consist of the energy-momentum tensor given in Eqs (3) and (4) and the line element expressed in Eq (2) is given in [56].

$$\begin{array}{ccc} 8\pi \rho +E^{2} & = & \frac{1}{r^{2}}-e^{-\lambda }(\frac{1}{r^{2}}+\frac{\mu_{,r}-\lambda_{,r}}{r}+\frac{\mu_{,r}^{2}-\mu_{,r}\lambda_{,r}}{4}+\frac{\mu_{,rr}}{2})\\ & - & e^{-\nu }(\frac{\mu_{,t}\lambda_{,t}}{4}), \end{array}$$ (7)

$$\begin{array}{ccc} -8\pi p+E^{2} & = & \frac{1}{r^{2}}-e^{-\lambda }(\frac{1}{r^{2}}+\frac{\nu_{,r}+\mu_{,r}}{r}+\frac{\mu_{,r}\nu_{,r}}{4})\\ & + & \frac{e^{-\nu }}{4}(2\mu_{,tt}+{\mu_{,t}}^{2}-\mu_{,t}\nu_{,t}), \end{array}$$ (8)

$$\begin{array}{ccc} 8\pi p+E^{2} & = & \frac{e^{-\nu }}{4}(2(\mu_{,tt}+\lambda_{,tt})+\lambda_{,t}(\lambda_{,t}-\nu_{,t})+\mu_{,t}(\mu_{,t}-\nu_{,t}+\lambda_{,t}))\\ & - & \frac{e^{-\lambda }}{4}\{(\nu_{,r}^{2}+\mu_{,r}^{2}-\nu_{,r}\lambda_{,r}+2(\nu_{,rr}+\mu_{,rr})-\mu_{,r}(\lambda_{,r}+\nu_{,r}))\\ & + & \frac{2}{r}(\nu_{,r}+\mu_{,r}-\lambda_{,r})\}, \end{array}$$ (9)

$$\begin{array}{ccc} 8\pi T_{1}^{0} & = & (\frac{\mu_{,r}\mu_{,t}}{4}-\frac{\mu_{,t}\nu_{,r}}{4}-\frac{\mu_{,r}\lambda_{,t}}{4}+\frac{\mu_{,tr}}{2}-\frac{\lambda_{,t}}{r}),\;\;\;\;\;\;\;\; \end{array}$$ (10)

and

$$\begin{array}{c} {\left[r^{2}E\right]}_{,r}=4\pi r^{2}\sigma e^{\left(\lambda +\mu \right)/2}. \end{array}$$ (11)

The subscripts *t* indicate the partial derivative with respect to time, while the subscripts *r* denote the partial derivative with respect to radius. Eq (11) can be expressed for the electric field E in the following equivalent form:

$$\begin{array}{c} E\left(r\right)=\frac{1}{r^{2}}\int_{0}^{r}4\pi r^{2}\sigma e^{\left(\lambda +\mu \right)/2}dr=\frac{q(r)}{r^{2}} \end{array}$$ (12)

## Five-dimensional CKV

A more useful formulation of the CKV that given in Eq (1) is as follows:

$$\begin{array}{c} L_{\xi }g_{mn}=\xi_{m;n}+\xi_{n;m}=\psi g_{mn},\;\;\;\;\;m,n=1,2,3,4,5. \end{array}$$ (13)

In this instance, *ξ* represents the orbit group and *ψ* represents the conformal factor. The metric *g*_*mn*_ is conformally transformed to itself while considering *ξ*_*i*_. Let’s also expand on the supposition that the group’s orbit is orthogonal to the vector field that represents the fluid’s velocity.

$$\begin{array}{c} \xi^{\mu }u_{\mu }=0. \end{array}$$ (14)

The following results from the spherical symmetry found in Eq (14):

$$\begin{array}{c} \xi^{1}=\xi^{3}=....=\xi^{n+1}=0. \end{array}$$

The CKV applied to the line element (2) provides the following set of equations:

$$\nu_{,r}\xi^{2}=\psi ,$$ (15)

$$\lambda_{,r}\xi^{2}+2\xi_{,2}^{2}=\psi ,$$ (16)

$$\xi^{2}=\frac{\psi r}{2},$$ (17)

$$\mu_{,r}\xi^{2}=\psi .$$ (18)

The result of the previous set of equations is

$$e^{\nu }=r^{2}C_{1}^{2},$$ (19)

$$e^{\lambda }=\frac{C_{2}^{2}}{\psi^{2}},$$ (20)

$$e^{\mu }=r^{2}C_{3}^{2},$$ (21)

where the integration constants are denoted by *C*_1_, *C*_2_, and *C*_3_. By substituting the EMFEs (7)–(9) with the equivalents of (19)–(21), we get:

$$8\pi \rho +E^{2}=\frac{1}{r^{2}}(1-\frac{3\psi^{2}}{C_{2}^{2}})-\frac{3\psi \psi_{,r}}{rC_{2}^{2}},$$ (22)

$$-8\pi p+E^{2}=\frac{1}{r^{2}}(1-\frac{6\psi^{2}}{C_{2}^{2}}),$$ (23)

$$8\pi p+E^{2}=-\frac{2\psi \psi_{,r}}{rC_{2}^{2}}+\frac{\psi^{2}}{r^{2}C_{2}^{2}}-\frac{2}{r^{2}}-\frac{\psi_{,r}}{r\psi }.$$ (24)

From the above Eqs (22)–(24), we can obtain the values of *E*, *ρ* and *p* [57].

$$\begin{array}{c} E^{2}=-\frac{\psi \psi_{,r}}{rC_{2}^{2}}-\frac{5\psi^{2}}{2r^{2}C_{2}^{2}}-\frac{1}{2r^{2}}-\frac{\psi_{,r}}{2r\psi }, \end{array}$$ (25)

$$\begin{array}{c} 8\pi \rho =\frac{3}{2r^{2}}-\frac{2\psi \psi_{,r}}{rC_{2}^{2}}-\frac{\psi^{2}}{2r^{2}C_{2}^{2}}+\frac{\psi_{,r}}{2r\psi }, \end{array}$$ (26)

$$\begin{array}{c} 8\pi p=-\frac{3}{2r^{2}}-\frac{\psi \psi_{,r}}{rC_{2}^{2}}+\frac{7\psi^{2}}{2r^{2}C_{2}^{2}}-\frac{\psi_{,r}}{2r\psi }. \end{array}$$ (27)

Note that the relationship between the metric potentials and the physical parameters *ρ* and *p* is significantly provided by Eqs (26) and (27):

$$\begin{array}{c} 8\pi \left(\rho +p\right)=\frac{3\psi^{2}}{r^{2}C_{2}^{2}}-\frac{3\psi \psi_{,r}}{rC_{2}^{2}}. \end{array}$$ (28)

The three dependent variables in the non-linear differential equation system above are *ρ*, *p*, and *ψ*, whereas the independent variable is *r*. Therefore, we have two different equations that involve the three unknowns *ρ*, *p*, and *ψ* in Eqs (26) and (27). As a result, we have two equations and three unknowns. To balance the equations, we will use the density profile, which is provided in [1] as

$$\begin{array}{c} \rho =\frac{1}{8\pi }\left(\frac{a}{r^{2}}+3b\right). \end{array}$$ (29)

Different star configurations are produced here by the constants *a* and *b*. Consequently, we obtain by changing Eq (29) for Eq (26).

$$\begin{array}{c} \frac{2}{5}\psi +\frac{4r}{3\psi }-\frac{1}{3}=\frac{2}{3\psi }\left(3-2a\right)C_{2}^{2}-bC_{2}^{2}\left(\frac{4r^{2}}{\psi }-2r-\frac{\psi }{5}\right), \end{array}$$ (30)

From Eq (30), we get

$$\begin{array}{ccc} \psi & = & \frac{1}{6(2+bC_{2}^{2})}(5+30brC_{2}^{2})\\ & - & \sqrt{{\left(-5-30brC_{2}^{2}\right)}^{2}-4\left(6+3bC_{2}^{2}\right)\left(-30C_{2}^{2}+20aC_{2}^{2}+20r+60bC_{2}^{2}r^{2}\right)}). \end{array}$$

## Exact analytical solution for the model

The exact analytical solution can be obtained as

$$\begin{array}{ccc} e^{\lambda } & = & C_{2}^{2}{\left(12+6bC_{2}^{2}\right)}^{2}(\left(5+30brC_{2}^{2}\right)\\ & - & \sqrt{{\left(-5-30brC_{2}^{2}\right)}^{2}-4\left(6+3bC_{2}^{2}\right)\left(-30C_{2}^{2}+20aC_{2}^{2}+20r+60bC_{2}^{2}r^{2}\right)}{)}^{-2}. \end{array}$$ (31)

The radial pressure is obtained as

$$\begin{array}{ccc} p & = & \frac{1}{8\pi }(\frac{-3}{2r^{2}}-\frac{30bC_{2}^{2}-\frac{\alpha }{2\beta }}{2r(5+30brC_{2}^{2}-\beta )}\\ & - & \frac{30bC_{2}^{2}-\frac{\alpha }{2\beta }}{36{\left(2+bC_{2}^{2}\right)}^{2}C_{2}^{2}r}\left(5+30brC_{2}^{2}-\beta \right)+\frac{{\left(5+30brC_{2}^{2}-\beta \right)}^{2}}{72{\left(2+bC_{2}^{2}\right)}^{2}C_{2}^{2}r^{2}}) \end{array}$$ (32)

where

$$\alpha =(-60bC_{2}^{2}\left(-5-30brC_{2}^{2}\right)-4\left(6+3bC_{2}^{2}\right)\left(20+120brC_{2}^{2}\right)),$$ (33)

$$\beta =\sqrt{{\left(-5-30brC_{2}^{2}\right)}^{2}-4\left(6+3bC_{2}^{2}\right)\left(-30C_{2}^{2}+20r+60br^{2}C_{2}^{2}+20aC_{2}^{2}\right)}.$$ (34)

## Higher dimensional exterior spacetime and anisotropy induced by electric charge

The exterior geometry associated with the higher-dimensional electromagnetic field in the context of Reissner-Nordström-Vaidya is studied in [58]

$$\begin{array}{c} ds^{2}=(1-\frac{M(u)}{R^{2}}+\frac{q^{2}}{3r^{4}})du^{2}+2dudR-R^{2}(d\theta_{1}^{2}+sin^{2}\theta_{1}(d\theta_{2}^{2}+sin\theta_{2}^{2}d\theta_{2}^{3})), \end{array}$$ (35)

When we apply the junction conditions at the *r* = *a*_5_ boundary, we get

$$\begin{array}{c} 1-\frac{m}{a_{5}^{2}}+\frac{q^{2}}{3a_{5}^{4}}=C_{2}^{2}a_{5}^{2}, \end{array}$$ (36)

and

$$\begin{array}{ccc} 1 & - & \frac{m}{a_{5}^{2}}+\frac{q^{2}}{3a_{5}^{4}}=\frac{1}{C_{2}^{2}{\left(12+6bC_{2}^{2}\right)}^{2}}\{\left(5+30brC_{2}^{2}\right)\\ & - & {\left({\left(-5-30brC_{2}^{2}\right)}^{2}-\left(24+12bC_{2}^{2}\right)\left(-30C_{2}^{2}+20aC_{2}^{2}+20r+60bC_{2}^{2}r^{2}\right)\right)}^{1/2}{\}}^{2}. \end{array}$$ (37)

The anisotropy induced by electric charge in higher dimension in a form equivalent to an anisotropic fluid

$$\begin{array}{ccc} p_{t} & = & \frac{1}{8\pi }(\frac{-3}{2r^{2}}-\frac{30bC_{2}^{2}-\frac{\alpha }{2\beta }}{2r(5+30brC_{2}^{2}-\beta )}\\ & - & \frac{30bC_{2}^{2}-\frac{\alpha }{2\beta }}{36{\left(2+bC_{2}^{2}\right)}^{2}C_{2}^{2}r}\left(5+30brC_{2}^{2}-\beta \right)+\frac{{\left(5+30brC_{2}^{2}-\beta \right)}^{2}}{72{\left(2+bC_{2}^{2}\right)}^{2}C_{2}^{2}r^{2}}), \end{array}$$ (38)

where *α* and *β* are defined in (33) and (34).

## Analysis of model

We shall now move forward with a comparative analysis of the physical characteristics. There are various techniques that can be used for this. But we’ll be concentrating on two key areas in this study. First and foremost, we will assess the stability of the models in higher dimensions. Second, in order to get additional understanding, we will investigate how charge varies with respect to different physical factors, including conformal parameter, metric potential, *ρ*, *p*, and Δ. The modified TOV equation is represented as

$$\begin{array}{c} (\frac{\nu_{,r}+\mu_{,r}}{2})\rho +p_{r}-\frac{dp_{r}}{dr}+\frac{2}{r}\left(p_{t}-p_{r}\right)=0. \end{array}$$ (39)

The above-mentioned TOV equation describes the star configuration’s equilibrium under the effects of gravity. The effects of gravity are described as *F*_*g*_, hydrostatic force *F*_*h*_, and anisotropic stress *F*_*a*_ and it can be represented as follows:

$$\begin{array}{c} F_{g}+F_{h}+F_{a}=0, \end{array}$$ (40)

where

$$F_{g}=-\frac{\left(\nu_{,r}+\mu_{,r}\right)}{2}\left(\rho +p_{r}\right),$$ (41)

$$F_{h}=-\frac{dp_{r}}{dr},$$ (42)

$$F_{a}=\frac{2}{r}\left(p_{t}-p_{r}\right).$$ (43)

### Energy conditions

First, let’s delve into a crucial aspect of stellar models. Understanding the energy conditions within the scope of GR is pivotal in studying CO. Here, we investigate three key energy conditions—(i) the Null Energy Condition (NEC), (ii) the Weak Energy Condition (WEC), and (iii) the Strong Energy Condition (SEC)—simultaneously held at all points within the star’s interior, outlined by the following inequalities:

- NEC: *ρ* ≥ 0,
- WEC: *ρ* + *p*_*r*_ ≥ 0, *ρ* + *p*_*t*_ ≥ 0,
- SEC: *ρ* + *p*_*r*_ + 2*p*_*t*_ ≥ 0.

Figs 1–8 display the model’s characteristics graphically.

**Fig 1:**
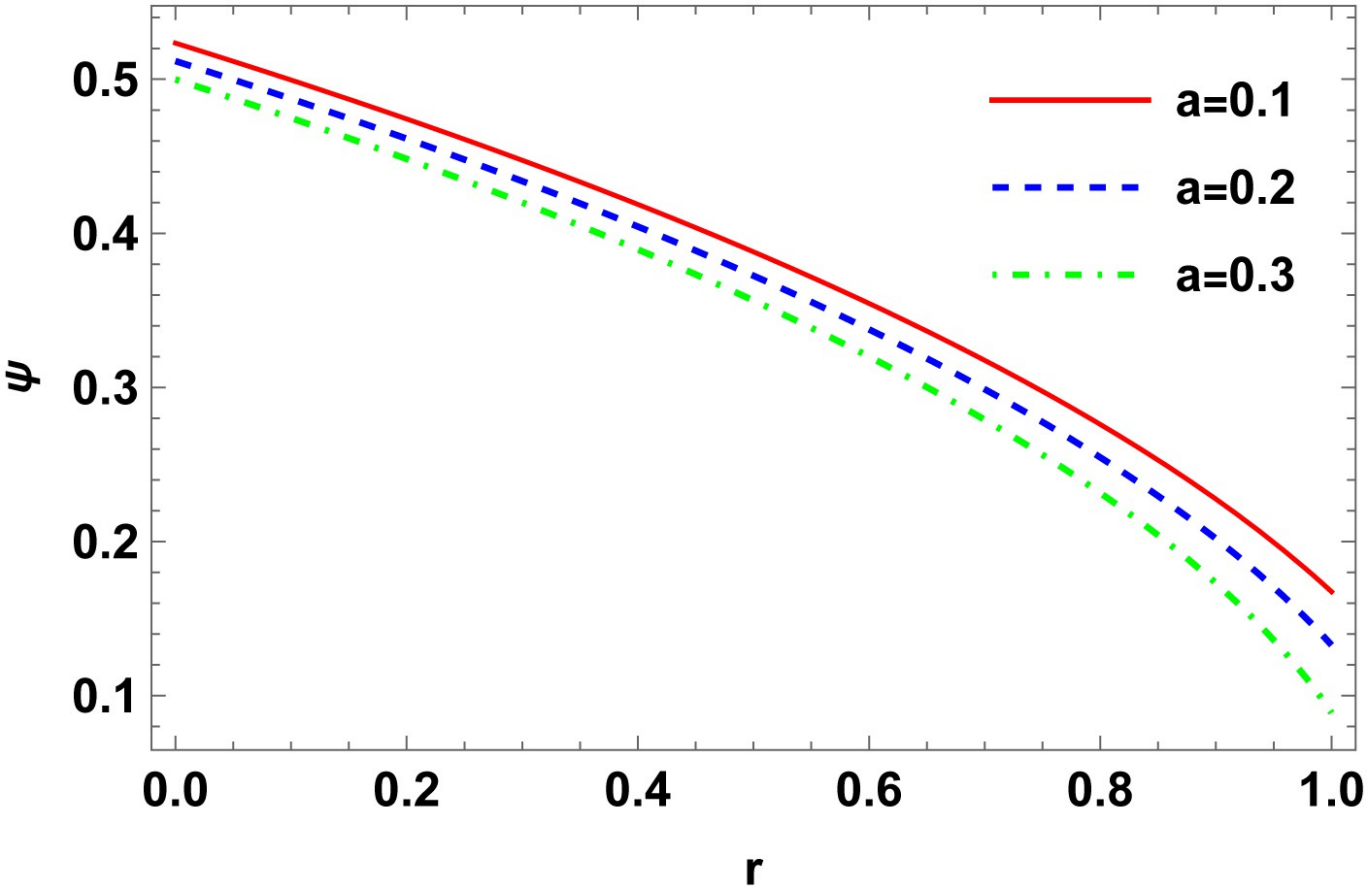
The variation of conformal factor (*ψ*) for E (*b* = 0.03, *C*_2_ = 0.1).

**Fig 2:**
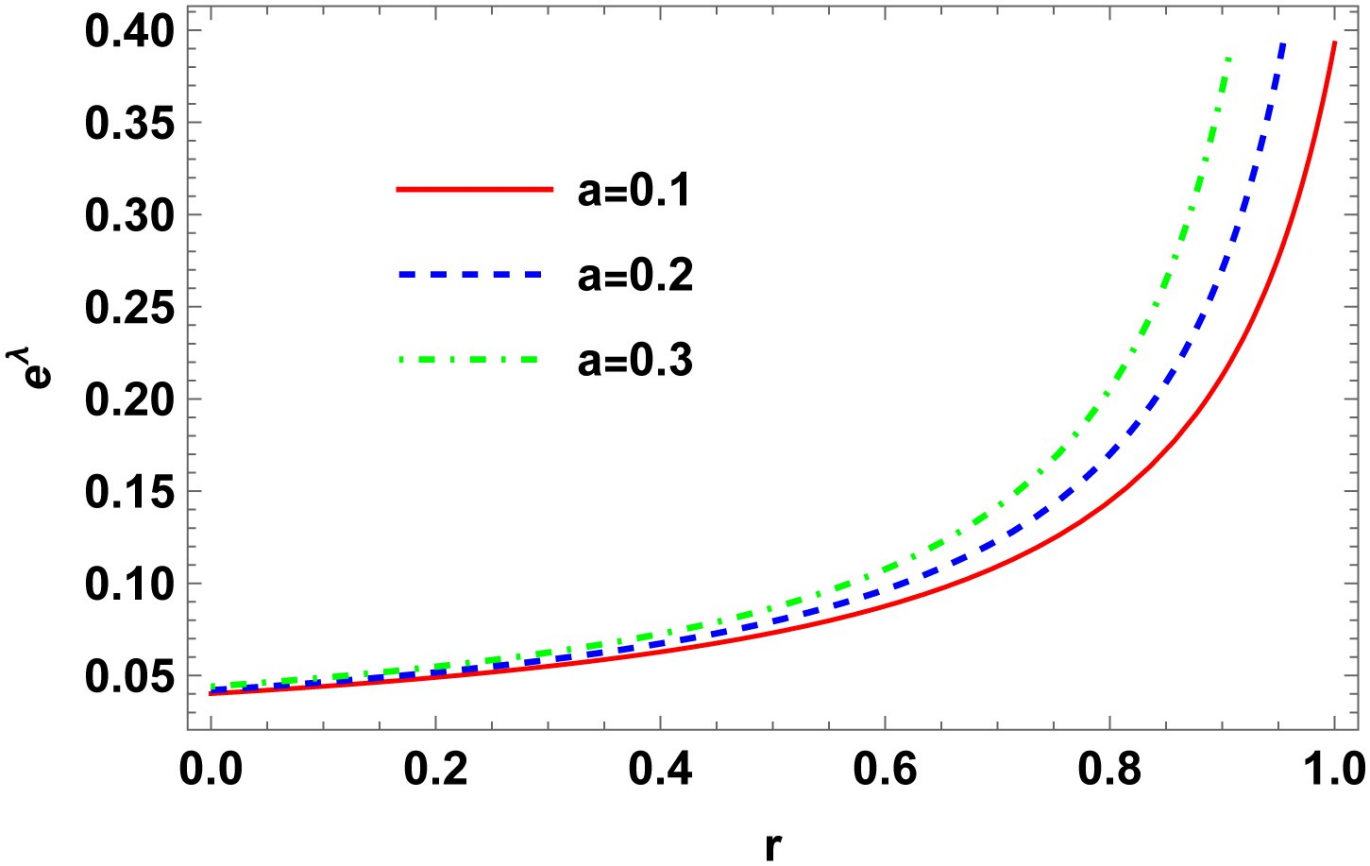
Variation of metric coefficient (*e*^λ^) for E (*b* = 0.03, *C*_2_ = 0.1).

**Fig 3:**
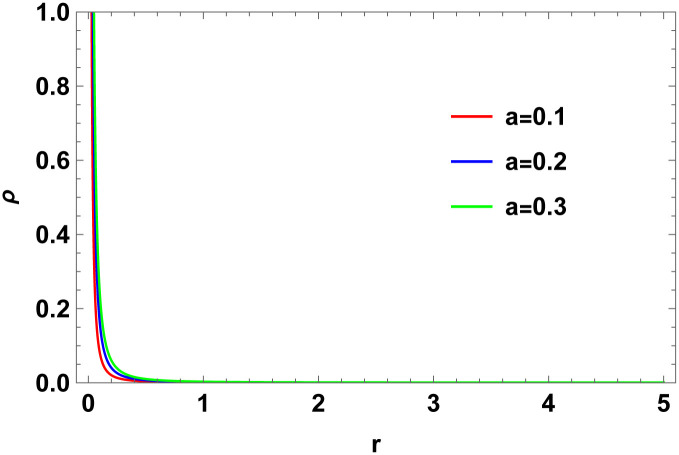
The density profile (*ρ*) for *b* = 0.03.

**Fig 4:**
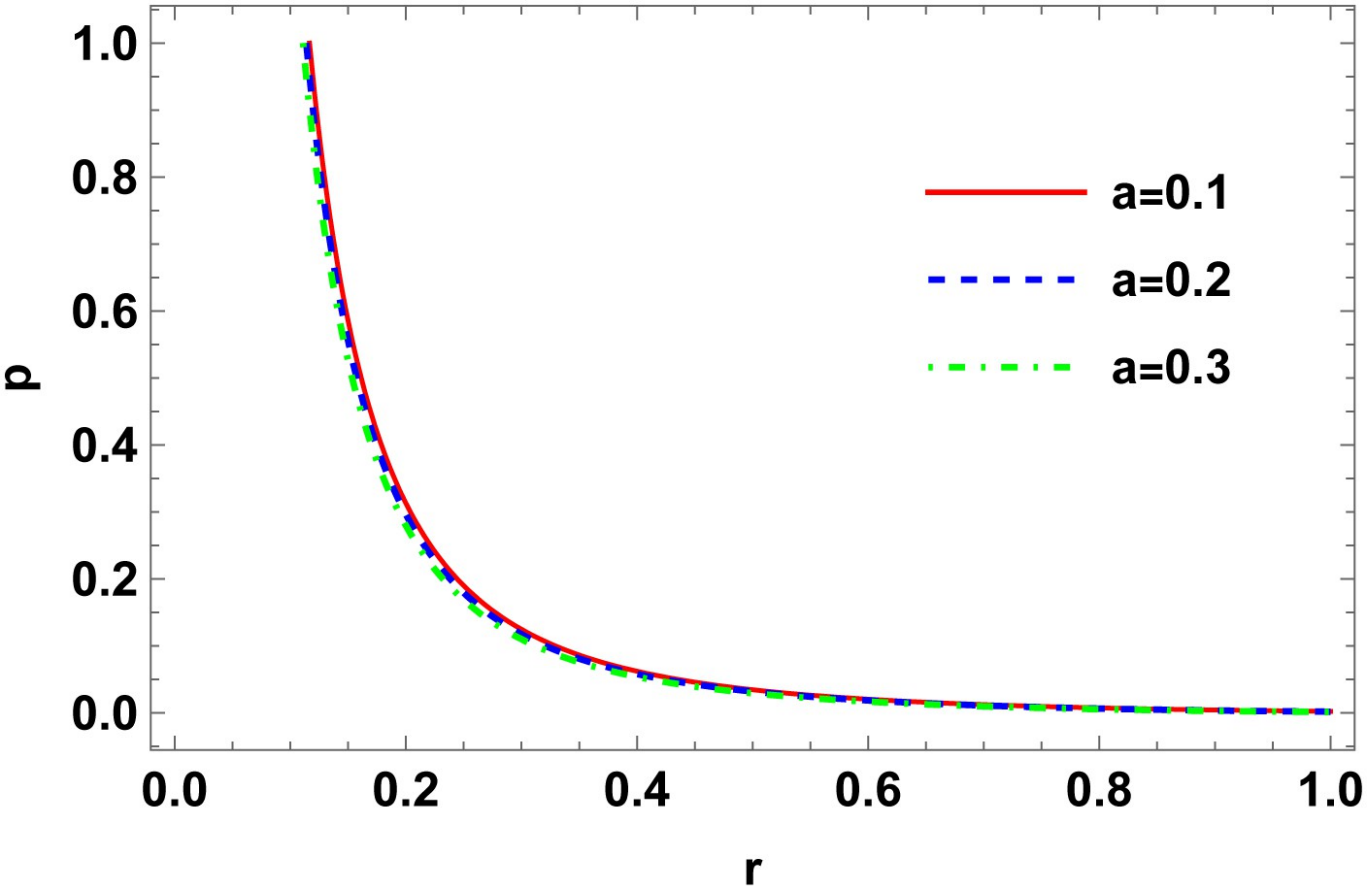
Variation of isotropic pressure for E (*b* = 0.03, *C*_2_ = 0.1).

**Fig 5:**
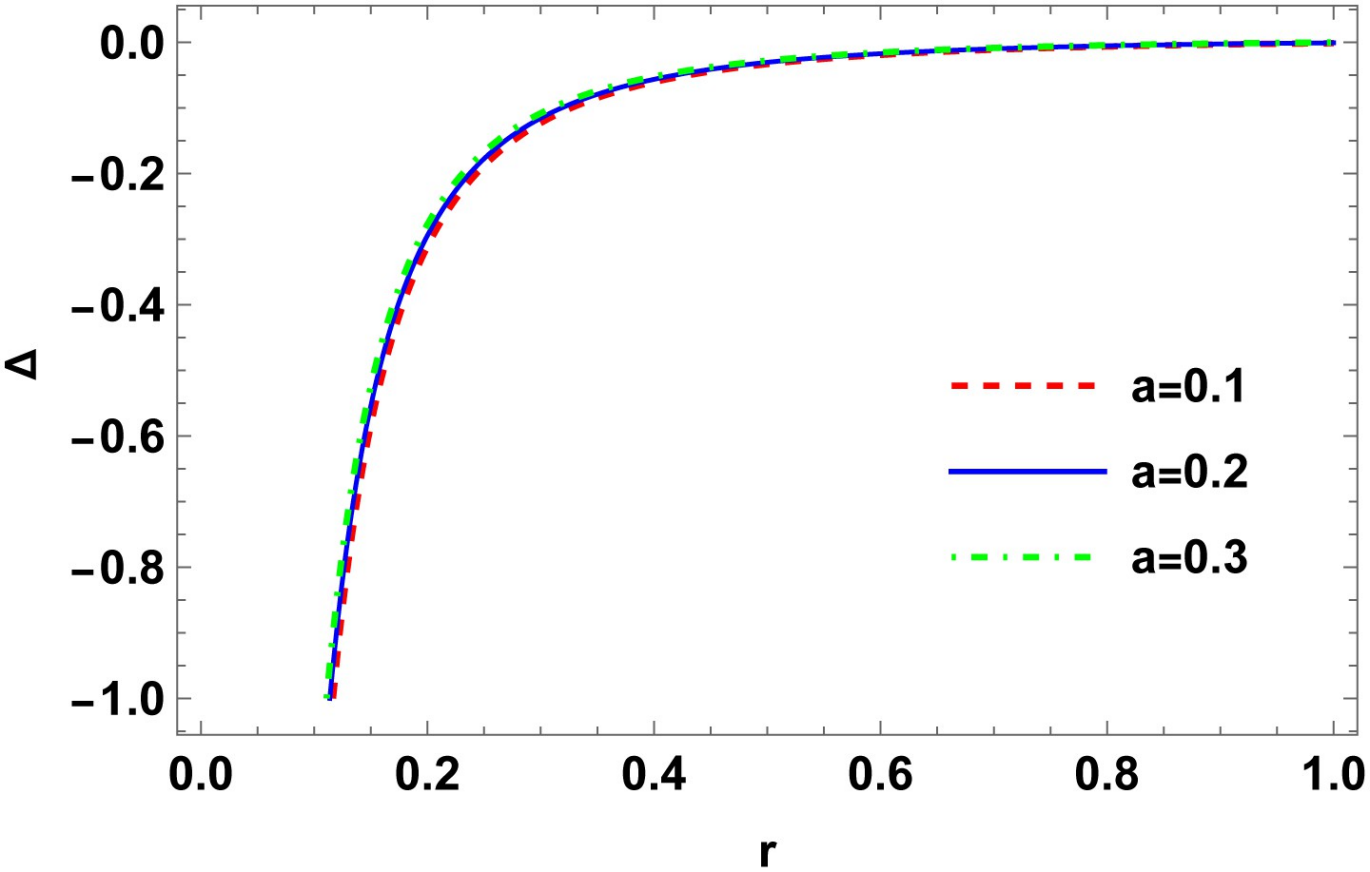
Anisotropy factor for E (*b* = 0.03, *C*_2_ = 0.1).

**Fig 6:**
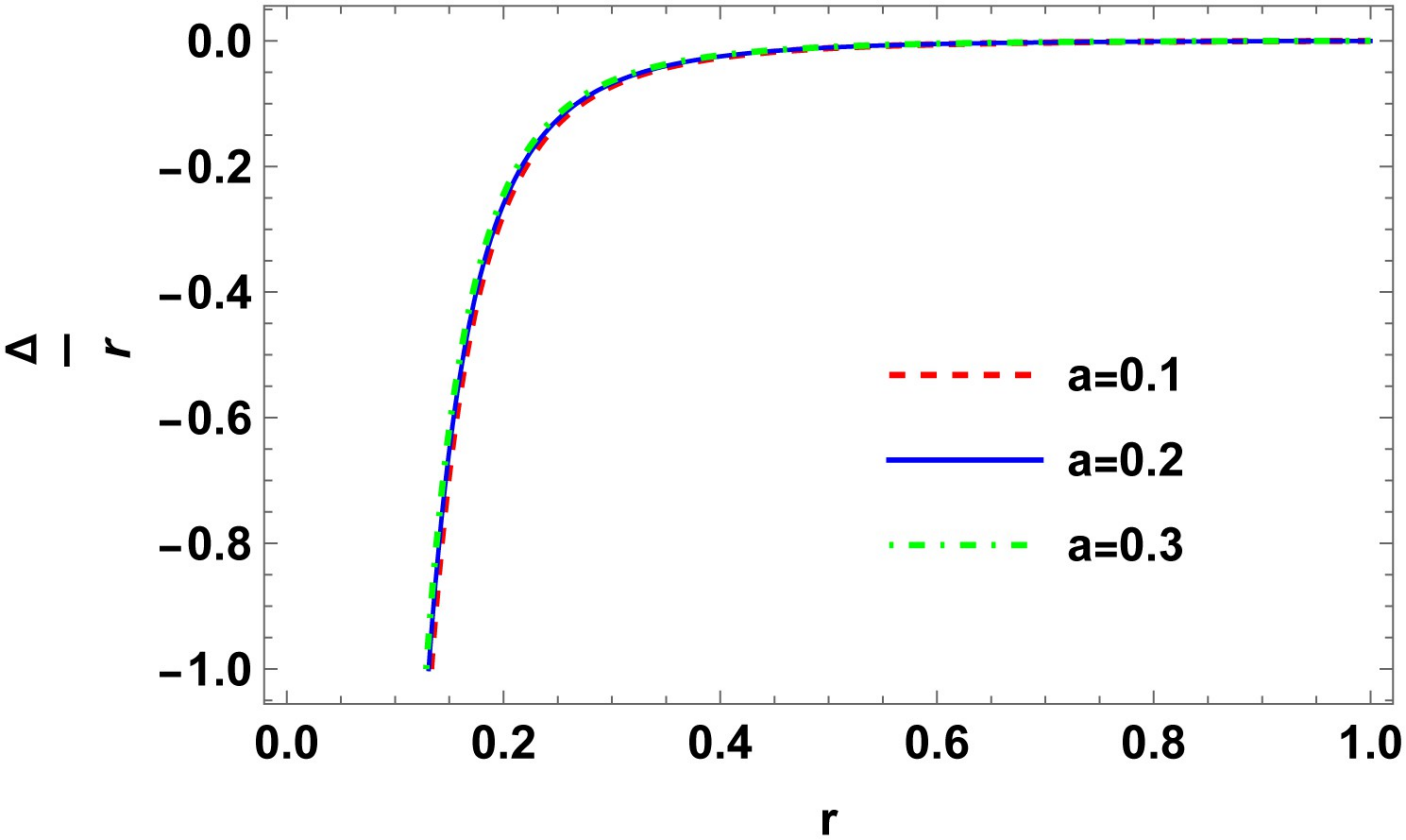
Variation of anisotropy force for E (*b* = 0.03, *C*_2_ = 0.1).

**Fig 7:**
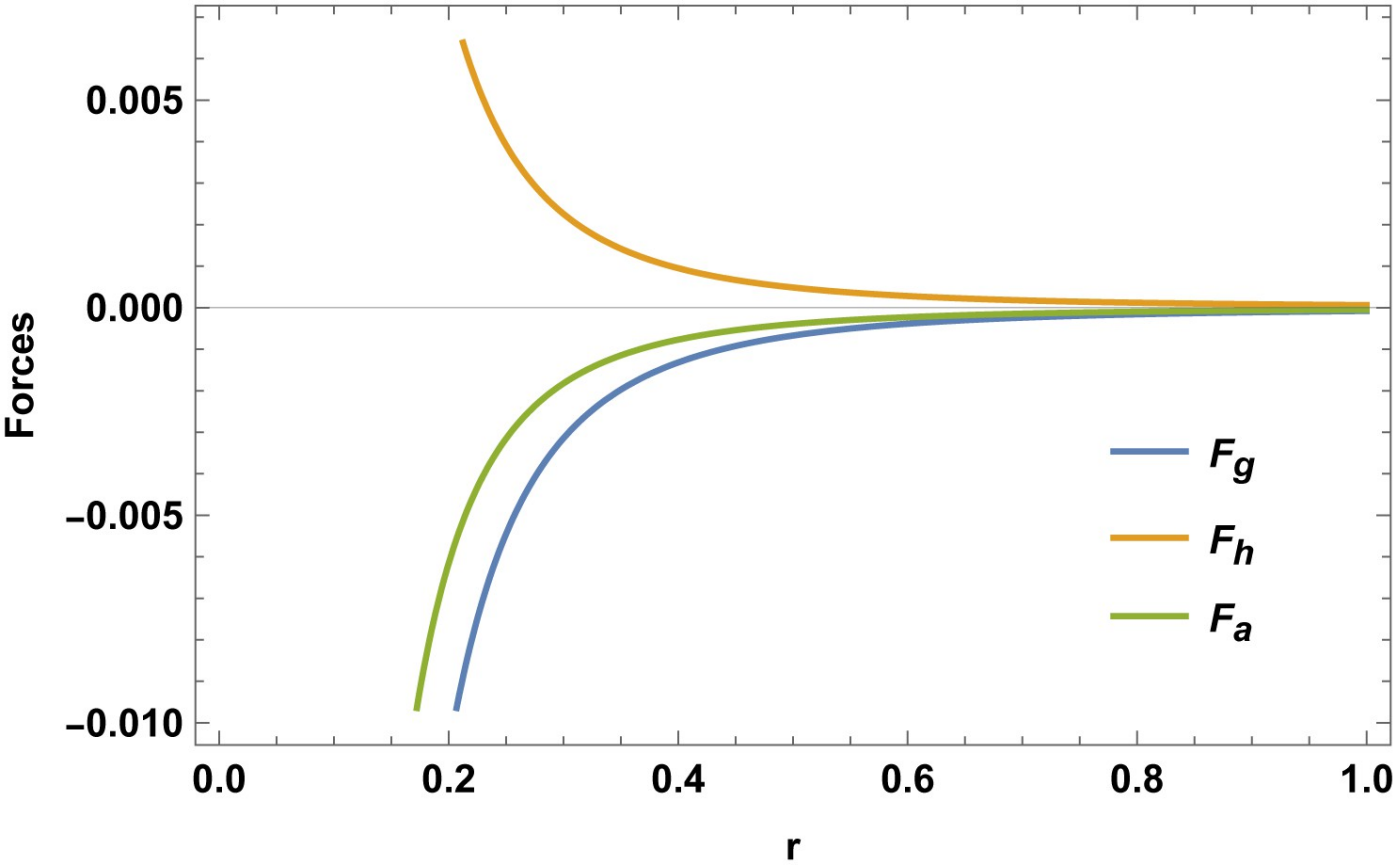
The three forces (*F*_*g*_, *F*_*h*_, *F*_*a*_) for E (*b* = 0.03, *C*_2_ = 0.1).

**Fig 8:**
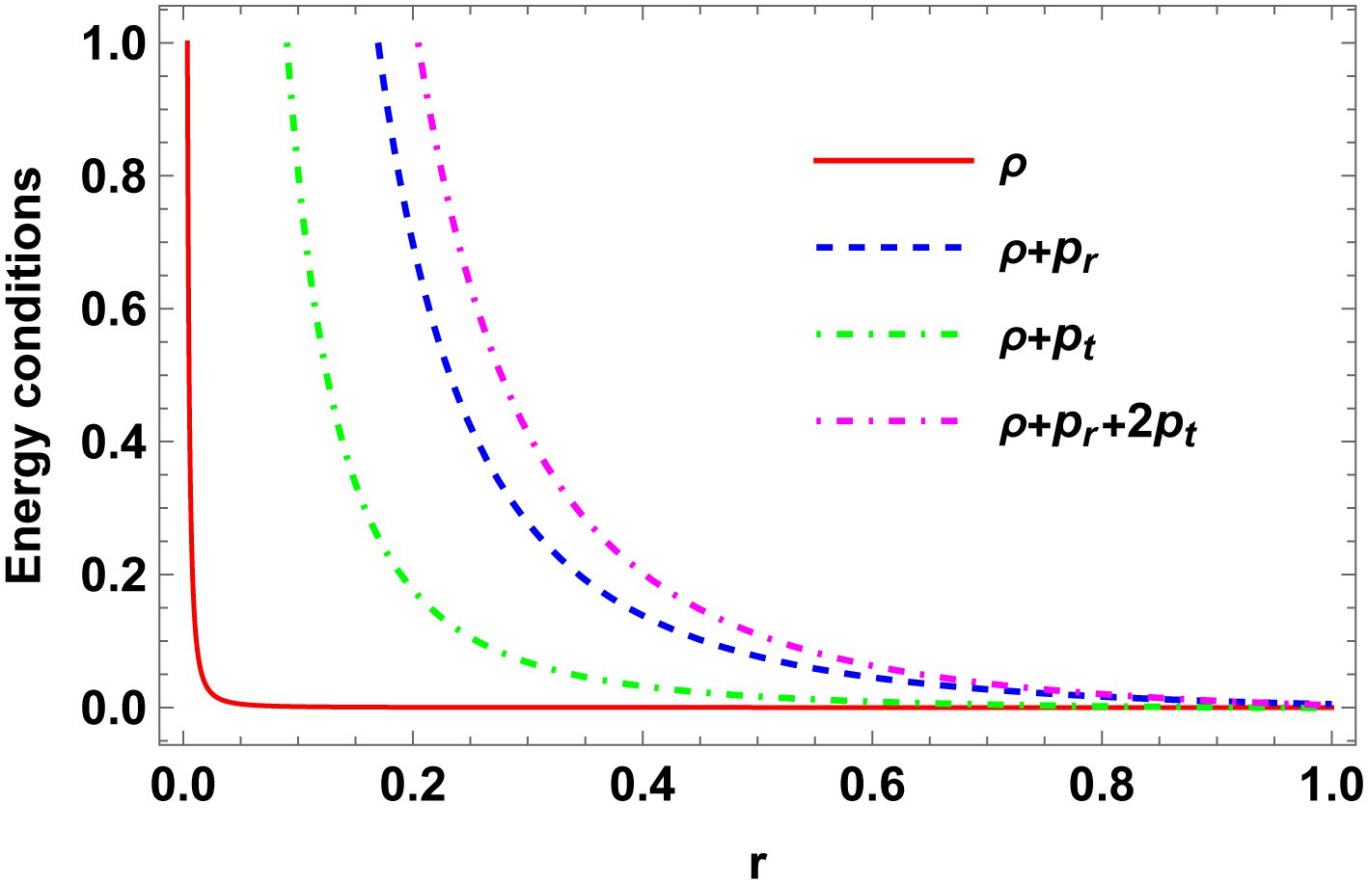
Energy conditions for E (*a* = 0.1, *a* = 0.2, *a* = 0.3, *b* = 0.03, *C*_2_ = 0.1).

## Summary and analysis

In this study, we investigated a new framework for isotropic charged sphere conformal motion in a five-dimensional non-static spherical system. This spacetime geometry’s EMFEs are to be solved using a certain density distribution function. Bhar [59] discussed about the higher dimensional charged gravastar admitting conformal motion. For this analysis, he considered static spherically symmetric spacetime in higher dimensions and found the mass of the thin shell. This study motivated us to develop the framework for charged COs admitting conformal motion in higher dimensions. We considered the dynamic spherically symmetric spacetime in five dimensions for anisotropic fluid distribution. The anisotropy induced due to electric charge in higher dimensioanl spacetime. Here, the pressure of the fluid is divided into two distinct components: transverse pressure and radial pressure. The dissimilarity between these values signifies the relationship between the stiffness of the star’s core and its surface tension. In this instance, we have introduced a novel analytical approach that describes charged CO that allow for conformal motion.

These well-defined and compact solutions are an excellent tool for deepening our understanding of the physical dynamics of charged, CO. Establishing a stable solution and gaining a deeper understanding requires examining the behaviours of fundamental physical parameters including density, stellar configuration equilibrium and forces. Figs 1–8 display the characteristics of the model’s behavior.

- Figs 1 and 2 illustrate variations in the conformal factor and metric potential, respectively.
- Fig 3 shows a positive characteristic and a stable configuration of the energy density.
- In Fig 4, the isotropic pressure exhibits a positive characteristic.
- The electrical charge-induced anisotropy in a sphere. The anisotropy is oriented inward if Δ > 0, and outward if Δ < 0. While the curve in Fig 5 demonstrates that both the anisotropic force and Δ < 0 are attractive in nature, the Δ > 0 suggests a repulsive anisotropic force.
- The anisotropic force due to the star’s nature is $\frac{\Delta }{r}$. This force is repulsive if $\frac{\Delta }{r}>0$; otherwise, it is attracting.
- The Fig 7 illustrates the three forces *F*_*g*_, *F*_*h*_, and *F*_*a*_ that are derived from the TOV equation for our specified source and contribute to system stability.
- The energy conditions depicted in Fig 8 are satisfied by the interior spacetime in five dimensions.

To analyze the stability of the general framework, we have opted generalized TOV equation and energy conditions. We have found that our framework satisfies all the necessary conditions. Based on the facts presented here, we could make assumptions about the existence of charged CO in higher dimensions. The characteristics of the model, including conformal factor, metric potential, energy conditions, pressure, and anisotropy, are examined using a specific density profile for five-dimensional spacetime. For the solution of charged higher dimensional spacetime, all of these parameters are well-behaved. The selected energy density distribution strongly demonstrates that CO exists in higher dimensions.

## References

[1] Rahaman F., et al., A class of solutions for anisotropic stars admitting conformal motion. Astrophys. Space Sci. 2010; 330: 249–56. doi: 10.1007/s10509-010-0384-0

[2] Aktas C., Yilmaz I., Charged anisotropic Bardeen spheres admitting conformal motion. Gen. Rel. Gravi. 2007; 39: 84962

[3] Rahaman F., et al., On role of pressure anisotropy for relativistic stars admitting conformal motion. Astrophys. Space. Sci. 2010; 325: 137–47. doi: 10.1007/s10509-009-0167-7

[4] Mak M. K., Harko T., Quark stars admitting a one-parameter group of conformal motions. Internat. J. Mod. Phys. D 2004; 13(1): 149–56. doi: 10.1142/S0218271804004451

[5] Esculpi M., Aloma E., Conformal anisotropic relativistic charged fluid spheres with a linear equation of state. Eur. Phys. J. C 2010; 67: 521–32. doi: 10.1140/epjc/s10052-010-1273-y

[6] Shamir M. F., Massive compact Bardeen stars with conformal motion. Phys. Lett. B 2020; 811: 135927. doi: 10.1016/j.physletb.2020.135927

[7] Lemos J. P. S., Winberg E. J., Quasiblack holes from extremal charged dust. Phys. Rev. D 2004; 69(10); 104004. doi: 10.1103/PhysRevD.69.104004

[8] Lemos J. P. S., Zanchin V. T., Quasiblack holes with pressure: relativistic charged spheres as the frozen stars. Phys. Rev. D 2010; 81(12): 124016. doi: 10.1103/PhysRevD.81.124016

[9] Bonnor W. B., Non-spherical quasi-black holes. Gen. Rel. Grav. 2010; 42: 1825–30. doi: 10.1007/s10714-010-0950-3

[10] Barreto W., Rodriguez B., Rosales L., Serrano O., Self-similar and charged radiating spheres: an anisotropic approach. Gen. Rel. Grav. 2007; 39: 23–39. doi: 10.1007/s10714-006-0365-3

[11] Ivanov B. V., The importance of anisotropy for relativistic fluids with spherical symmetry. Int. J. Theor. Phys. 2010; 49: 1236–43. doi: 10.1007/s10773-010-0305-6

[12] Thirukkanesh S., Maharaj S. D., Charged anisotropic matter with a linear equation of state. Class. Quantum Grav. 2008; 25(23): 235001. doi: 10.1088/0264-9381/25/23/235001

[13] Böhmer C. G., Harko T., Lobo F.S.N., Conformally symmetric traversable wormholes. Phys. Rev. D 2007; 76(8): 084014. doi: 10.1103/PhysRevD.76.084014

[14] Böhmer C. G., Harko T., Lobo F.S.N., Wormhole geometries with conformal motions. Class. Quantum. Gravit. 2008; 25(7): 075016. doi: 10.1088/0264-9381/25/7/075016

[15] Shabbir G., Hussain F., Kara A. H., Ramzan M., A note on some perfect fluid Kantowski–Sachs and Bianchi type III spacetimes and their conformal vector fields in f(R) theory of gravity. Mod. Phys. Lett. A 2019; 34(11): 1950079. doi: 10.1142/S0217732319500792

[16] Herrera L., Jimenez J. et al., Anisotropic fluids and conformal motions in general relativity. J. Math. Phys. 1984 25(11): 3274–78. doi: 10.1063/1.526075

[17] Herrera L., Ponce de Leon J., Perfect fluid spheres admitting a one-parameter group of conformal motions. J. Math. Phys. 1985; 26(4): 778–784. doi: 10.1063/1.526567

[18] Herrera L., Ponce de Leon J., Anisotropic spheres admitting a one-parameter group of conformal motions. J. Math. Phys. 1985 26(8): 2018–23. doi: 10.1063/1.526872

[19] Herrera L., Ponce de Leon J., Isotropic and anisotropic charged spheres admitting a one-parameter group of conformal motions. J. Math. Phys. 1985; 26(9): 2302–7. doi: 10.1063/1.526813

[20] Herrera L., Ponce de Leon J., Confined gravitational fields produced by anisotropic fluids. J. Math. Phys. 26(11). doi: 10.1063/1.526710

[21] Mason D. P., Maartens R., Kinematics and dynamics of conformal collineations in relativity. J. Math. Phys. 1987; 28(9): 2182–86. doi: 10.1063/1.527431

[22] Di Prisco A., Herrera L., Jimenez J. et al., The Bondi metric and conformal motions. J. Math. Phys. 1987; 28(11): 2692–96. doi: 10.1063/1.527713

[23] Saridakis E., Tsamparlis M., Symmetry inheritance of conformal Killing vectors. J. Math. Phys. 1991; 32(6): 1541–51. doi: 10.1063/1.529263

[24] Di Prisco A., Herrera L., Esculpi M., Self-similar scalar soliton stars in the thin-wall approximation. Phys. Rev. D 1991; 44(8): 2286. doi: 10.1103/PhysRevD.44.2286 10014110

[25] Aguirregabiria J. M., Di Prisco A., Herrera L., Ibanez J., Time evolution of self-similar scalar soliton stars: A general study. Phys. Rev. D 1992; 46(6): 2723. doi: 10.1103/PhysRevD.46.2723 10015205

[26] Yavuz I., Yilmaz I., Baysal H., Strange quark matter attached to the string cloud in the spherical symmetric space–time admitting conformal motion. Int. J. Mod. Phys. D 2005; 14(08): 1365–72. doi: 10.1142/S0218271805007061

[27] Herrera L., Di Prisco A., Self-similarity in static axially symmetric relativistic fluids. Int. J. Mod. Phys. D 2018: 27(01): 1750176. doi: 10.1142/S0218271817501760

[28] Herrera L., Di Prisco A., Ospino J., Non-static fluid spheres admitting a conformal Killing vector: exact solutions. Universe. 2022; 8(6): 296. doi: 10.3390/universe8060296

[29] Duggal K. L., Sharma R., Conformal collineations and anisotropic fluids in general relativity. J. Math. Phys. 1986; 27(10): 2511–13. doi: 10.1063/1.527317

[30] Ray S., Usmani A. A., Rahaman F., Kalam M., Chakraborty K., Electromagnetic mass model admitting conformal motion. arXiv preprint arXiv:0806.3568. 2008

[31] Pradhan S., Sahoo P. K., A comprehensive study of massive compact star admitting conformal motion under bardeen geometry. Nucl. Phys. B 2024; 1002: 116523. doi: 10.1016/j.nuclphysb.2024.116523

[32] Rahaman F., Ray S., Kalam M. et al., Do Solar system tests permit higher dimensional general relativity?. Int. J. Theor. Phy. 2009; 48: 3124–38. doi: 10.1007/s10773-009-0110-2

[33] Liu H., Overduin J. M., Solar system tests of higher dimensional gravity. Astrophys. J. 2000; 538(1): 386. doi: 10.1086/309115

[34] Rahaman F., Chakraborty S., Ray S., et al., The higher dimensional gravastars. Int. J. Theor. Phy. 2015; 54: 50–61. doi: 10.1007/s10773-014-2198-2

[35] Rahaman F., Pradhan A., Ahmed N., et al., Fluid sphere: Stability problem and dimensional constraint. Int. J. Mod. Phys. 2015; 24(7): 1550049. doi: 10.1142/S0218271815500492

[36] Zahra A., Mardan S. A., Riaz M. B., Conformal motion for higher-dimensional compact objects. Eur. Phys. J. C 2023; 83(12): 1107 (2023) doi: 10.1140/epjc/s10052-023-12289-x

[37] Bhar P., Rahaman F., Ray S., Chatterjee V., Possibility of higher-dimensional anisotropic compact star. Eur. Phys. J. C. 2015; 75(5): 190. doi: 10.1140/epjc/s10052-015-3375-z

[38] Chattopadhyay P. K., Deb R., Paul B. C., Relativistic Charged Star Solutions in Higher Dimensions. Int. J. Theo. Phys. 2014; 53: 1666–84. doi: 10.1007/s10773-013-1965-9

[39] Dey S., Paul B. C., Higher dimensional charged compact objects in Finch–Skea geometry. Classical. Quant. Grav. 2020; 37(7): 075017. doi: 10.1088/1361-6382/ab75ae

[40] Clayton D. D., Principles of Stellar Evolution and Nucleosynthesis. University of Chicago Press, Chicago 1983.

[41] Kippenhahn R., Weigert A., Stellar Structure and Evolution. Springer, Berlin 1991.

[42] Glendenning N. K., Compact Stars: Nuclear Physics, Particle Physics and General Relativity. Springer, Berlin 1997.

[43] Ruderman M., Pulsars: structure and dynamics. Annu. Rev. Astron. Astrophys. 1972; 10: 427. doi: 10.1146/annurev.aa.10.090172.002235

[44] Herrera L. and Santos N. O., Local anisotropy in self-gravitating systems. Phys. Report. 1997; 286(2): 53–130. doi: 10.1016/S0370-1573(96)00042-7

[45] Dev K., Gleiser M., Anisotropic stars: exact solutions. Gen. Relativ. Gravit. 2002; 34; 1793–1818. doi: 10.1023/A:1020707906543

[46] Bhar P., Malik A., Almas A., Impact of f(Q) gravity on anisotropic compact star model and stability analysis. Chin. J. Phys. 2024; 88: 839–56. doi: 10.1016/j.cjph.2024.02.016

[47] Rashid A., Malik A., Shamir M. F., A comprehensive study of Bardeen stars with conformal motion in f(G) gravity. Eur. Phy. J. C 2023; 83(11): 997. doi: 10.1140/epjc/s10052-023-12141-2

[48] Asghar Z., Malik A., Shamir M. F., Mofarreh F., Comprehensive analysis of relativistic embedded class-I exponential compact spheres in *f*(*R*, *ϕ*) gravity via Karmarkar condition. Comm. Theo. Phys. 2023; 75(10): 105401. doi: 10.1088/1572-9494/acf123

[49] Asghar Z., Shamir M. F., Usman A., Malik A, Study of embedded class-I fluid spheres in *f*(*R*, *T*) gravity with Karmarkar condition. Chin. J. Phys. 2023; 83: 427–37. doi: 10.1016/j.cjph.2023.04.009

[50] Naz T., Malik A., Ramay Z., Quasinormal Modes of Dilaton Black Holes: Analytic Approximations. Int. J. Theo. Phys. 2024; 63(5): 1–14.

[51] Naz T., Malik A., Saleem H., Waheed S., Finch-Skea Stellar structures obeying Karmarkar condition in modified *f*(*G*) gravity. Chin. J. Phys. 2024; 89: 871–83. doi: 10.1016/j.cjph.2024.03.037

[52] Naz T., Malik A., Gillani D., Mofarreh F., Relativistic configurations of Tolman stellar spheres in *f*(*G*, *T*) gravity. Int. J. Geom. Methods Mod. Phys. 2023; 20(13): 2350222. doi: 10.1142/S0219887823502225

[53] Naz T., Malik A., Asif M. K., Fayyaz I., Evolving embedded traversable wormholes in f (R, G) gravity: a comparative study. Phys. Dark Universe. 2023; 42: 101301. doi: 10.1016/j.dark.2023.101301

[54] Yousaf Z., Bhatti M. Z., Aman H., Malik A., Bouncing cosmology with 4D-EGB gravity. Int. J. Theo. Phys. 2023; 62(7): 155. doi: 10.1007/s10773-023-05409-6

[55] Millward R. S., A five-dimensional Schwarzschild-like solution. Gen. Relativ. Quantum. Cosm. 2006; arXiv:gr-qc/0603132.

[56] Zahra A., Mardan S. A. and Noureen I., Analysis of heat flow in the post-quasi-static approximation for gravitational collapse in five dimension. Eur. Phys. J. C 2023; 83(!): 51. doi: 10.1140/epjc/s10052-023-11383-4

[57] A Usmani A., Rahaman F., Ray S. et al., Charged gravastars admitting conformal motion. Phys. Lett. B 2011; 701(4): 388–92. doi: 10.1016/j.physletb.2011.06.001

[58] Zahra A., Mardan S. A. Five dimensional analysis of electromagnetism with heat flow in the post-quasi-static approximation. Eur. Phys. J. C 2023; 83(3): 1–9. doi: 10.1140/epjc/s10052-023-11383-4

[59] Bhar P., Higher dimensional charged gravastar admitting conformal motion. Astrophys. Space. Sci. 2014; 354: 457–62. doi: 10.1007/s10509-014-2109-2

